# Isolation and identification of flavonoids components from *Pteris vittata* L.

**DOI:** 10.1186/s40064-016-3308-9

**Published:** 2016-09-23

**Authors:** Li-jing Lin, Xiao-bing Huang, Zhen-cheng Lv

**Affiliations:** 1 Agricultural Product Processing Research Institute, Chinese Academy of Tropical Agricultural Sciences, Zhanjiang, 524001 China; 2 Department of Life Science, Huizhou University, Huizhou, 516007 China

**Keywords:** *Pteris vittata* L., Flavonoids, Kaempferol-3-*O*-d-glucopyranoside

## Abstract

**Background:**

*Pteris vittata* L. is rich in flavonoids which exhibit different bioactivities. In order to investigate the flavonoids components of *P. vittata* L., extracts of the plant were isolated by column chromatography on silica gel and Sephadex LH-20.

**Results:**

Four flavonoids compounds were obtained, the structures were identified as kaempferol (**1**), quercetin (**2**), kaempferol-3-*O*-d-glucopyranoside (**3**) and rutin (**4**), respectively, on the basis of NMR spectroscopic analyses.

**Conclusions:**

Compound **3** was reported for the first time from *P. vittata* L.

**Electronic supplementary material:**

The online version of this article (doi:10.1186/s40064-016-3308-9) contains supplementary material, which is available to authorized users.

## Background

*Pteris vittata* L., a common fern known as ‘Chinese Brake Fern’, is native from China and widespread all over the world (Ma et al. 2001). It received much attentions in recent years because it was known to be a hyperaccumulator plant of arsenic used in phytoremediation (Cesaro et al. 2015; Tisarum et al. 2015; de Oliveira et al. 2016; Tiwari et al. 2016). It is also widely used in traditional Chinese medicine for diverse therapeutic applications, such as the treatment of influenza, dysentery, rheumatism, injury and scabies (Xie 1996). Previous qualitative phytochemical screening studies on *P. vittata* L. have showed a substantial amount of flavonoids (Ding et al. 2009; Zhou et al. 2010). Compounds like leucocyanidin, leucodelphinidin, flavone ester apigenin 7-*O*-*p*-hydroxybenzoate, as well as a number of glycosides of apigenin, leutolin, isocutellarein-8-*O*-methyl-ether, kaempherol and quercetin were isolated from this plant in the past years (Salatino and Prado 1998; Imperato 2006). But, it attracts little attention on its chemical constituents or bioactivities recently. Numerous reports showed that flavonoids exhibited different bioactivities, such as anti-inflammatory, anti-oxidative, hypolipidemic, or antitumor effects (Bao et al. 2016; Feng et al. 2014; Matias et al. 2014; Raman et al. 2016). Plants are one of the important sources for screening active compounds. Hence, we attempt to obtain flavonoids components from *P. vittata* L. to provide more information about its chemical constituents in this experiment.

## Methods

### Plant material

The whole plant of *P. vittata* L. was collected from Guangzhou, China, in October 2015 and identified by Prof. P. T. Li (College of Forestry, South China Agricultural University, Guangzhou, P. R. China). The voucher specimen (No. PGZ090239) has been deposited at the CANT Herbarium, South China Agricultural University, Guangzhou, P. R. China.

### Experimental material

Silica gel (100–200 and 200–300 mesh) for column chromatography (CC) and GF_254_ silica gel for thin layer chromatography (TLC) were purchased from Qingdao Marine Chemical Ltd., Qingdao, China. Sephadex LH-20 obtained from Amersham Biosciences, Sweden was used for CC. Other reagents were of analytical grade purchased from Guangzhou reagent Co. Ltd. NMR spectra (^1^H, ^13^C-NMR) were determined on a Bruker AV-600 instrument using TMS as an internal reference.

### Extraction and isolation

The whole plant (7.0 kg) of *P. vittata* L. was extracted with methanol for three times at 25 °C. After the solvent was removed under vacuum, the concentrated extract was further extracted successively with petroleum ether, ethyl acetate and *n*-butanol. The ethyl acetate extract (80 g) was subjected to silica gel column chromatography using gradient hexane–EtOAc (50:1–1:1) to obtain 6 fractions. Fraction 2 (12 g) was subjected to silica gel column chromatography eluted with gradient hexane–acetone (100:1–10:1) to afford compound **1** (52 mg). Fraction 3 (8 g) was subjected repeatedly to silica gel column chromatography eluted with hexane–acetone (100:5) and was finally purified by column chromatography on Sephadex LH-20 eluted with CHCl_3_–MeOH (1:1) to give compound **2** (53 mg). Fraction 5 (2 g) was subjected to silica gel column chromatography eluted with CHCl_3_–MeOH (95:5) to give compound **3** (36 mg). Fraction 6 (5 g) was subjected to silica gel column chromatography eluted with CHCl_3_–MeOH (10:1) led to the isolation of compound **4** (280 mg).

## Results

**Fig. 1:**
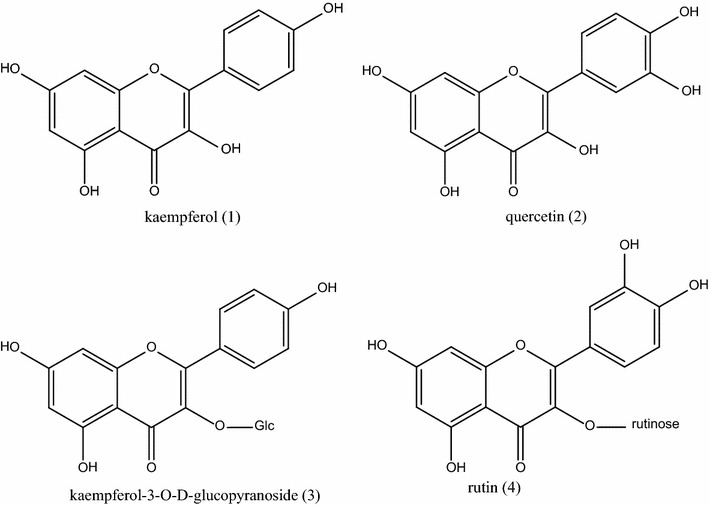
Structures of flavonoids from *P. vittata* L

Four compounds were isolated and their structures were identified as kaempferol (**1**), quercetin (**2**), kaempferol-3-*O*-d-glucopyranoside (**3**) and rutin (**4**), respectively, by spectral analysis and comparison with the spectroscopic data reported in previous literatures (Fig. 1). NMR spectra data of the compounds were listed as follows (Additional file 1).

Compound **1** was obtained as yellow amorphous powder. ^1^H-NMR (600 MHz, DMSO-*d*_*6*_) δ_H_: 6.19 (1H, d, *J* = 1.8 Hz), 6.44 (1H, d, *J* = 1.8 Hz), 6.92 (2H, d, *J* = 9.0 Hz), 8.04 (2H, d, *J* = 9.0 Hz). ^13^C-NMR (150 MHz, DMSO-*d*_*6*_) δ_C_: 93.5 (C-8), 98.2 (C-6), 103.0 (C-10), 115.4 (C-3′, C-5′), 121.7 (C-1′), 129.5 (C-2′), 130.5 (C-6′), 135.6 (C-3), 146.8 (C-2), 156.2 (C-5), 159.2 (C-4′), 160.7 (C-9), 163.9 (C-7), 175.9 (C-4). It was determined as kaempferol by comparison with the spectroscopic data reported in the literature (Liu et al. 2009).

Compound **2** was obtained as yellow amorphous powder. ^1^H-NMR (600 MHz, DMSO-*d*_*6*_) δ_H_: 6.19 (1H, d, *J* = 1.8 Hz), 6.40 (1H, d, *J* = 2.4 Hz), 6.88 (1H, d, *J* = 8.4 Hz), 7.54 (1H, dd, *J* = 2.4, 8.4 Hz), 7.67 (1H, d, *J* = 2.4 Hz). ^13^C-NMR (150 MHz, DMSO-*d*_*6*_) δ_C_: 93.3 (C-8), 98.1 (C-6), 102.9 (C-10), 114.9 (C-2′), 115.5 (C-5′), 119.9 (C-6′), 121.8 (C-1′), 135.6 (C-3), 144.9 (C-1′), 146.7 (C-1), 147.6 (C-4′), 156.1 (C-5), 160.7 (C-9), 163.8 (C-7), 175.8 (C-4). It was identified as quercetin by comparison with the spectroscopic data reported in the literature (Ma et al. 2007).

Compound **3** was obtained as yellow amorphous powder. ^1^H-NMR (600 MHz, DMSO-*d*_*6*_) δ_H_: 3.09 (2H, d, *J* = 4.2 Hz), 3.21 (1H, d, *J* = 7.8 Hz), 3.32 (1H, d, *J* = 11.4 Hz), 3.55 (1H, d, *J* = 11.4 Hz), 4.29 (1H, s), 4.96 (1H, s), 5.07 (1H, s), 5.36 (1H, s), 5.46(1H, d, *J* = 7.8), 6.21 (1H, d, *J* = 1.8 Hz), 6.43 (1H, d, *J* = 1.8 Hz), 6.88 (2H, d, *J* = 9.0 Hz), 8.04 (2H, d, *J* = 9.0 Hz). ^13^C-NMR (150 MHz, DMSO-*d*_*6*_) δ_C_: 61.1 (C-6″), 70.2 (C-4″), 74.5 (C-2″), 76.8 (C-5″), 77.8 (C-3″), 94.0 (C-8), 99.1 (C-6), 101.2 (C-1″), 104.3 (C-10), 115.4 (C-3′, C-5′), 121.2 (C-1′), 131.2 (C-2′, C-6′), 133.5 (C-3), 156.6 (C-2), 156.7 (C-9), 160.3 (C-4′), 161.5 (C-5), 164.5 (C-7), 177.8 (C-4). It was determined as kaempferol-3-*O*-d-glucopyranoside by comparison with the spectroscopic data reported in the literature (Long et al. 2011).

Compounds **4** was obtained as yellow amorphous powder. ^1^H-NMR (600 MHz, DMSO-*d*_*6*_) *δ*_H_: 1.00 (3H, d, *J* = 6.0 Hz), 3.08 (3H, m), 3.70 (1H, d, *J* = 12.0 Hz), 4.39 (1H, d, *J* = 2.4 Hz), 5.10 (2H, d, *J* = 18.0 Hz), 5.28 (1H, s), 5.35 (1H, d, *J* = 6.0 Hz), 6.20 (1H, d, *J* = 2.4 Hz), 6.39 (1H, d, *J* = 2.4 Hz), 7.54 (1H, d, *J* = 2.4 Hz), 7.56 (1H, dd, *J* = 2.4, 12.0 Hz) ^13^C-NMR (150 MHz, DMSO-*d*_*6*_): *δ*_C_: 17.6 (C-6′″), 66.9 (C-6″), 68.1 (C-5′″), 69.9 (C-4″), 70.2 (C-2′″), 70.4 (C-3′″), 71.7 (C-4′″), 73.9 (C-2″), 75.8 (C-5″), 76.3 (C-3″), 93.5 (C-8), 98.5 (C-6), 100.6 (C-1′″), 101.0 (C-1″), 103.8 (C-10), 115.1 (C-2′), 116.1 (C-5′), 121.0 (C-6′), 121.5 (C-1′), 133.2 (C-3), 144.6 (C-3′), 148.3 (C-4′), 156.3 (C-9), 156.5 (C-2), 161.1 (C-5), 164.0 (C-7), 177.2 (C-4). It was identified as rutin compared with the spectroscopic data reported in the literature (Zhang et al. 2010).

## Conclusion

Phytochemical screening of *P. vittata* L. was carried out. Four flavonoids were obtained and identified as kaempferol (**1**), quercetin (**2**), kaempferol-3-*O*-d-glucopyranoside (**3**) and rutin (**4**), respectively. Compound 1, 2 and 4 were known components which were mentioned in the previous results (Imperato and Telesca 2000; Imperato 2006). Compound **3** was a derivative of kaempferol with a glucopyranoside. It is reported for the first time from *P. vittata* L. The current results may provide more information about flavonoids profiles of this plant.

## Additional file

10.1186/s40064-016-3308-9 NMR spectra data of the compounds can be found online as Additional file for the present article.
