# Supplementary material for: Isolation and identification of flavonoids components from Pteris vittata L
Source: Springerplus. 2016 Sep 23;5(1):1649. doi: 10.1186/s40064-016-3308-9 (PMC5033795; doi:10.1186/s40064-016-3308-9)
Supplement: Supplementary file 1 — 10.1186/s40064-016-3308-9 NMR spectra data of the compounds can be found online as Additional file for the present article. [file 40064_2016_3308_MOESM1_ESM.docx]

**Supplementary Material**

**Isolation and Identification of flavonoids** **components from** ***Pteris vittata* L.**

Li-jing Lin^1^, Xiao-bing Huang^1^, Zhen-cheng Lv^2*^

1. Agricultural Product Processing Research Institute of Chinese Academy of Tropical Agricultural Sciences, Zhanjiang 524001, China;

2. Department of Life Science, Huizhou University, Huizhou 516007, China.

* Corresponding author. Tel.: +86-752-2529555; fax: +86-752-2529489

E-mail address: szsky@126.com


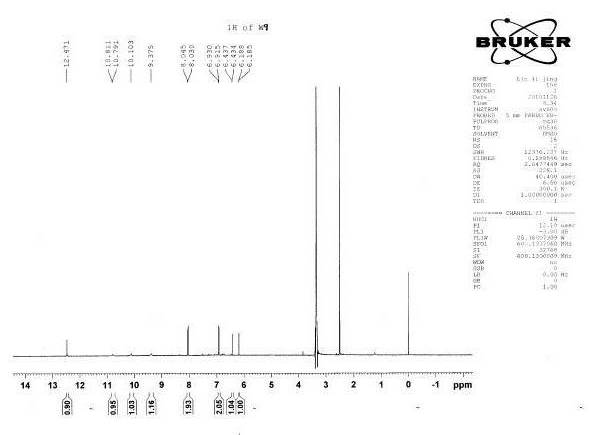


Fig. 1 ^1^H NMR（600 MHz，DMSO-*d6*）spectrum of compound 1


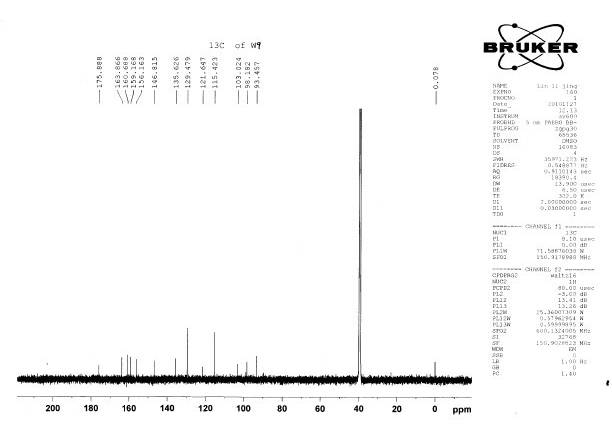


Fig. 2 ^13^C NMR（150 MHz，DMSO-*d6*）spectrum of compound 1


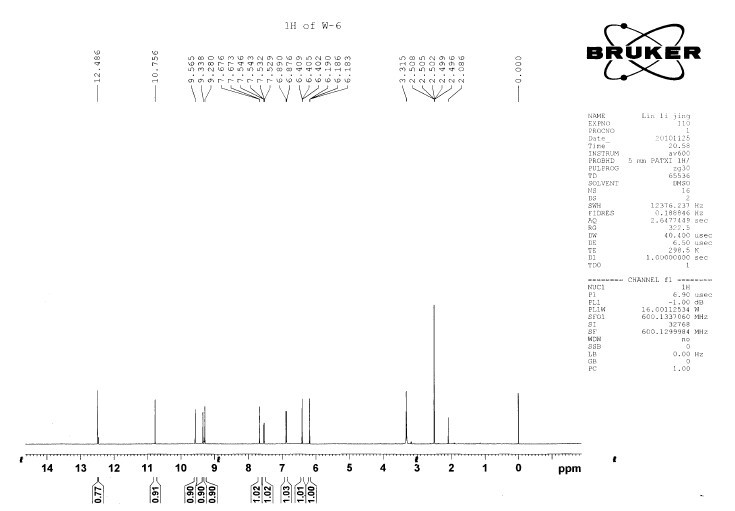


Fig. 3 ^1^H NMR（600 MHz，DMSO-*d6*）spectrum of compound 2


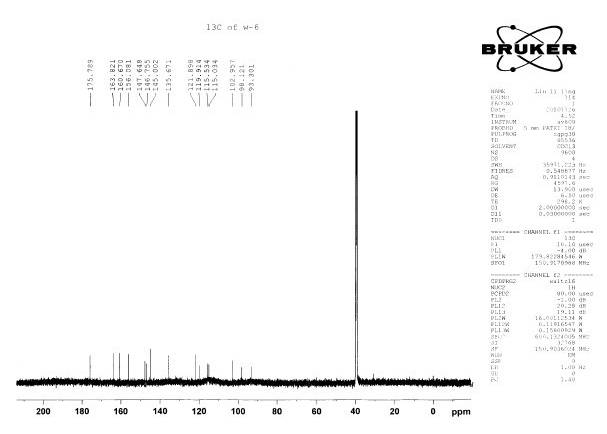


Fig. 4 ^13^C NMR（150 MHz，DMSO-*d6*）spectrum of compound 2

**
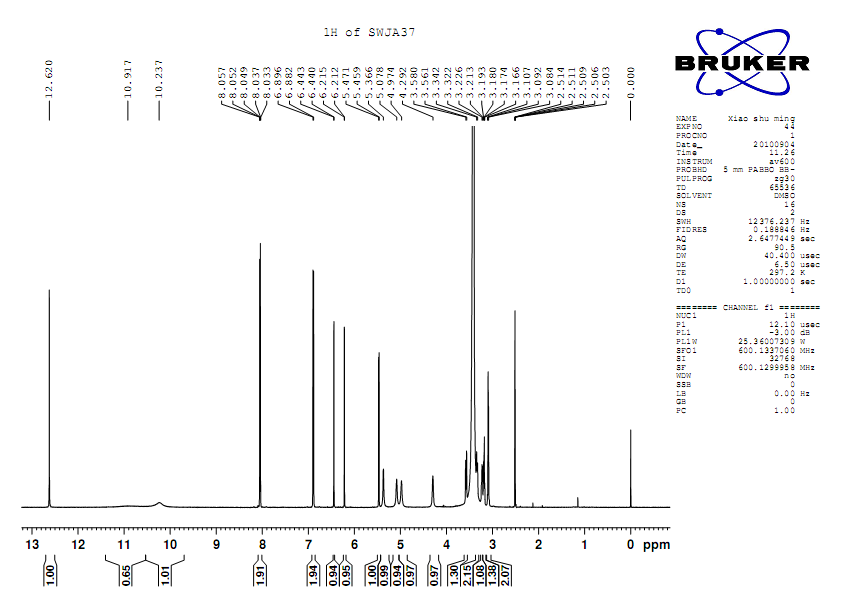
**

Fig. 5 ^1^H NMR（600 MHz，DMSO-*d6*）spectrum of compound 3

**
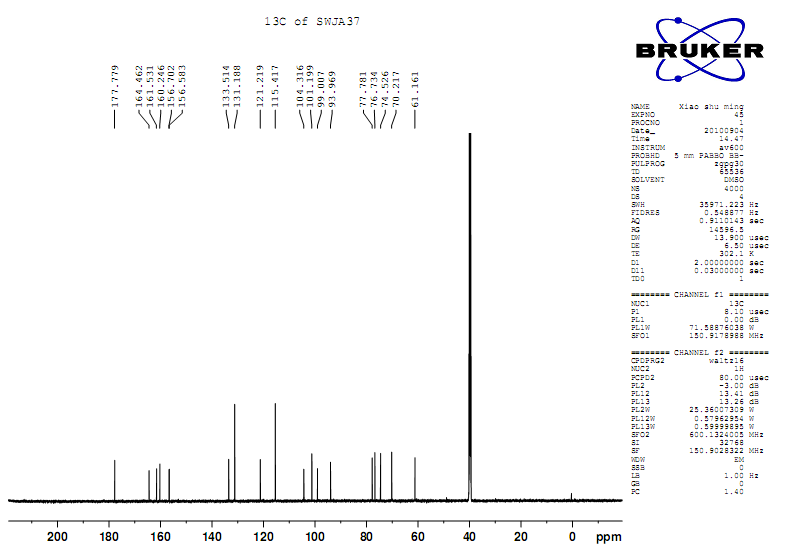
**

Fig. 6 ^13^C NMR（150 MHz，DMSO-*d6*）spectrum of compound 3


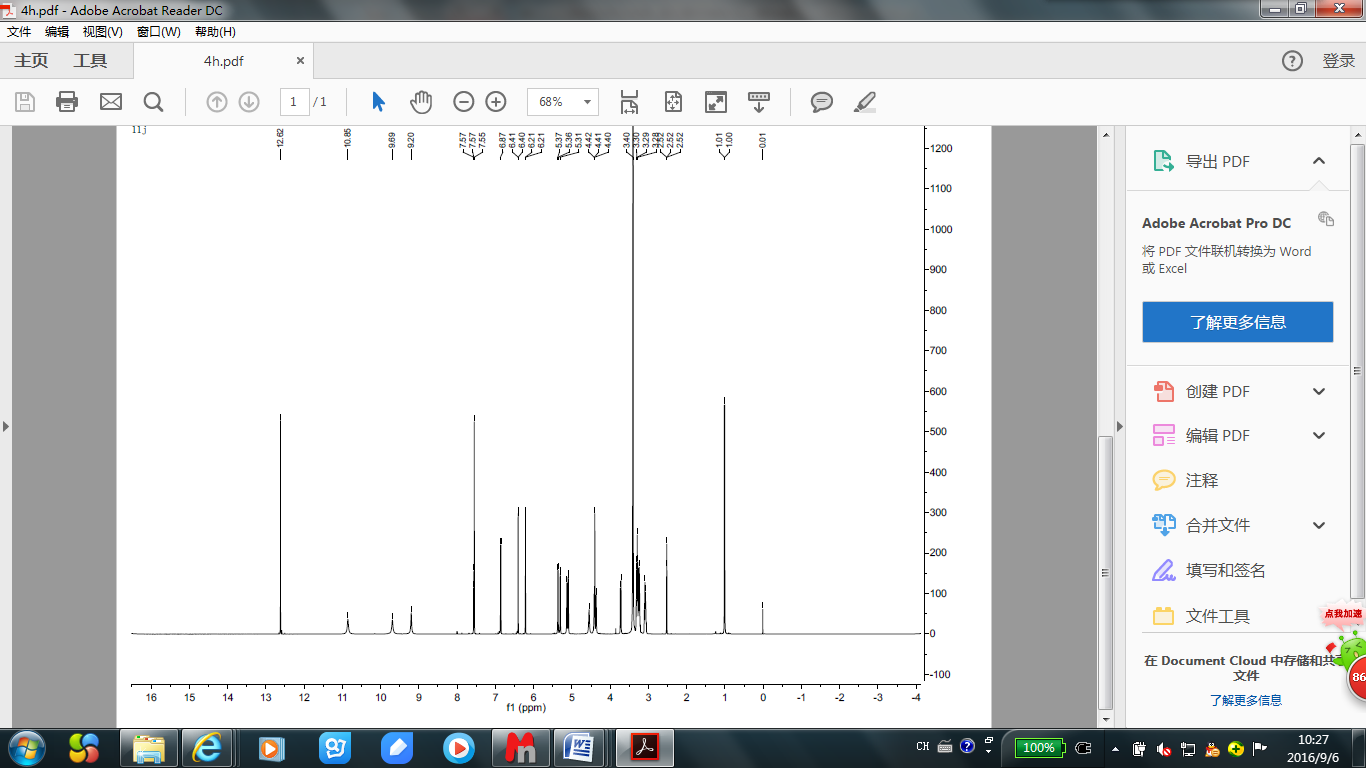


Fig. 7 ^1^H NMR（600 MHz，DMSO-*d6*）spectrum of compound 4


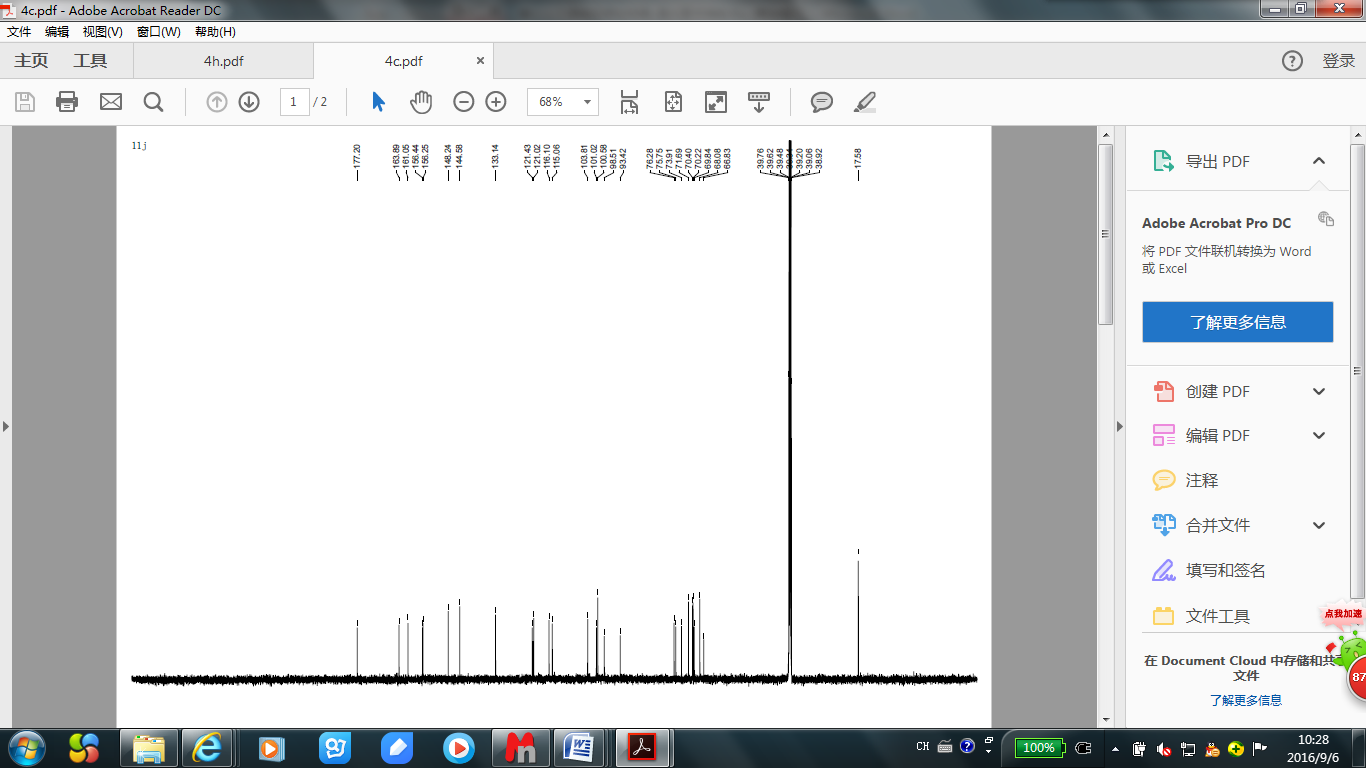


Fig. 8 ^13^C NMR（150 MHz，DMSO-*d6*）spectrum of compound 4
